# In-Depth Method Investigation for Determination of Boron in Silicate Samples Using an Improved Boron–Mannitol Complex Digestion Method by Inductively Coupled Plasma Mass Spectrometry

**DOI:** 10.3390/molecules28010441

**Published:** 2023-01-03

**Authors:** Xijuan Tan, Ruili Zhou, Yonggang Feng, Ting Liang

**Affiliations:** 1 Laboratory of Mineralization and Dynamics, Chang’an University, 126 Yanta Road, Xi’an 710054, China; 2 College of Earth Sciences and Land Resources, Chang’an University, 126 Yanta Road, Xi’an, 710054, China

**Keywords:** boron determination, silicate samples, boron–mannitol complex, wet acid method, ICP-MS

## Abstract

In this paper, a boron–mannitol complex wet acid digestion method proposed for the accurate determination of boron in silicate samples by inductively coupled plasma mass spectrometry (ICP-MS) was investigated in detail for the first time. With the addition of 50 μL of mannitol (2% wt.) into the mixture of 0.6 mL of concentrated HF and 30 μL of concentrated HNO_3_, the 50 mg of silicate sample was effectively decomposed after being heated overnight with optional pre-ultrasonic treatment. Following fluoride formation prevention by 8% HNO_3_ (wt.) and fluoride decomposition using 6% HCl (wt.), the samples were fluxed in 2.0 mL of 40% HNO_3_ (wt.) for 4 h and aged overnight. By diluting 1000-fold using 2% HNO_3_ (wt.) solution, the samples were directly quantified by an ICP-MS, showing boron recoveries of the standard materials including diabase W-2, basalt JB-2a, and rhyolite JR-2 in the range of 95.5–105.5% (*n* = 5). For this wet acid method, it was found that the contents of boron had no obvious difference under digestion temperatures of 65, 100, and 140 °C. It was also found that the ICP-MS quantification accuracy deteriorated at the mass of ^11^B when boron content was about 7250 ng yielding positive bias with average recoveries of 115.5–119.8% (*n* = 5), while the determination results remained unaffected at the mass of ^10^B. Furthermore, the digestion efficiency of boron by laboratory high-pressure closed digestion method was assessed. The boron recoveries with samples treated by the high-pressure closed digestion method were found to vary within 49.5–98.0% (*n* = 5) and even lowered down to 31.1% when skipping pressure relief procedure. The long-term quantification stability study showed that the boron content generally declined in one month for the high-pressure closed digestion method and exhibited no significant changes for the proposed method. By applying such an improved boron–mannitol complex digestion method, the boron concentration in the studied silicate standard materials were accurately determined, providing critical data for further boron isotope analyses and associated geochemical studies. This in-depth method investigation for silicate boron determination demonstrates the feasibility of this boron–mannitol complex strategy under a wide digestion temperature of 65–140 °C, and also sheds light on the extensive applications of boron as a geological tracer.

## 1. Introduction

Boron is a moderately to highly soluble and mobile element which has low atomic mass and two stable isotopes (i.e., ^10^B and ^11^B) [1,2]. This volatile, lithophile nonmetal element is strongly incompatible in most magmatic environments, while can be highly soluble in aqueous fluids when temperature is over 100 °C [3]. Thus, boron contents vary among different geological matrices [4,5]. Such properties make this element remarkably useful as a geochemical tracer for the growth of continental crust, the provenance identification of samples, and associated geological activities, such as the transfer and mixing processes both at the Earth’s and planetary sciences [6,7,8].

Boron concentration generally increases from basic to acid silicate samples [9]. For example, boron content in tourmaline, an acid rock-forming mineral, is larger than 3.0%. However, the basic basalt rocks such as datolite show boron to be higher than 6.0%, and the lowest boron in rock samples can be 0.5 μg/g. It is a fact that rock samples are characterized by containing boron contents in a wide range of variation [10]. To determine boron in silicate rocks, it is necessary to release boron from the silicate structure as in silicate structures Al^3+^ can be substituted by B^3+^ in tetrahedral coordination. Hydrofluoric acid (HF) and hydrochloric acid (HCl) are commonly utilized for silicate structure decomposition. However, the high volatility in forms of BF_3_ and BCl_3_ with respective boiling points of −100 and 12.5 °C leads to an easy escape of boron from samples even at room temperature [11]. This causes poor recovery of boron, making accurate quantification of boron and further isotope analysis challenging in geochemical studies. Among various boron digestion methods [12], despite boron in silicates being rendered chemically active to form borax by alkali fusion with sodium hydroxide or potassium carbonate [13,14,15], the direct analysis of trace boron is usually limited by the excessively complex matrix. Furthermore, the subsequent extraction of boron for isotope analysis by methyl-borate distillation or ion-exchange chromatography suffers from insufficient recovery and potential contaminations [16,17].

It is known that boric acid can react with a series of hydroxy compounds such as alcohol or phenol to form stable complexes [18]. Mannitol (C_6_H_14_O_6_), an easily accessible and non-toxic organic compound that contains six hydroxy groups, has been reported to be capable of forming boric-mannitol complex and thus suppressing the volatilization loss of boron from a HF or HCl solution [19,20]. Such a derivative complex may enable boron determination in silicate samples when applying HF and HCl as decomposition reagents. Nakamura et al. [21] successfully digested silicate samples with boron of 500 ng–5 μg by the mixture of mannitol and HF in an ultrasonic bath, which was followed by heating at 80 °C. However, a large quantity of about 0.3–5 mL of 1% mannitol solution was required. Additionally, complete sample dissolution was assessed by visual inspection and the produced fluoride residues were discarded. Makishima et al. [22] also applied mannitol-HF mixture to decompose rock samples in an ultrasonic bath. Less than 50 mg of rock powder was digested by 0.2 mL of 1% mannitol and 0.8 mL of 30 mol/L of HF. After recovering boron into 0.5 mol/L of HF solution, the fluoride residues were removed before boron determination. D’Orazio [23] reported a similar method by Nakamura et al. [21] to extract boron and achieved boron measurement from 1.35 to 157 μg/g in a matrix of about 1.98% HCl (*v*/*v*) by inductively coupled plasma mass spectrometry (ICP-MS). But the yielded precisions in relative standard deviations (RSDs) varied between 0.9% and 9.8%. 

Recently, Liu et al. [24] proposed a low-temperature acid-dissolution method for silicate samples containing 80–2000 ng of boron based on the usage of mannitol, giving nearly 100% recoveries for boron. However, this developed method required an ultrasonic treatment at least 4 h to assist sample decomposition and the temperature of all heating and evaporation steps in subsequent digestion process should be carefully controlled at 65 °C with a variation less than ±1.0 °C. Therefore, a low temperature of 65–80 °C was widely acceptable as a prerequisite for reasonable recoveries of boron analysis in silicate samples when using the boron–mannitol complex strategy. To the best of our knowledge, there has been no study of heating temperature effect on the recoveries of boron in silicate samples. 

In this current work, a wet acid digestion method using a ternary mixture of mannitol-HF-HNO_3_ was proposed for boron quantification in silicate samples. The boron recovery of this wet acid method under heating temperature in the range of 65–140 °C was studied in detail. The effect of ultrasonic treatment on sample digestion efficiency was also assessed. By applying this improved boron–mannitol complex wet acid method, boron contents in a series of geological standard materials from basic to acid compositions were successfully quantified by ICP-MS. Finally, this developed method was compared to the laboratory high-pressure closed digestion method [25] in terms of boron recovery and long-term quantification stability of boron.

## 2. Results and Discussion

### 2.1. Assessment of Boron Recovery of the Boron–Mannitol Complex Strategy

Since the binary acid mixture of HF-HNO_3_ has been proven to efficiently decompose silicate samples [25,26], this acid combination was coupled with mannitol yielding a ternary mixture as the digesting reagent in this work. To reduce fluorides formed during digestion, a prevention step for fluoride formation was introduced using a diluted HNO_3_ solution of 8% (wt.). Despite such fluorides being found to be boron free [21,27], 6% HCl solution (wt.) was added to decompose any produced fluorides with subsequent sample redissolution by using 40% HNO_3_ solution (*v*/*v*), which excluded further separation of sample solution from the insoluble matrix and thus avoided element loss due to coprecipitation. Here, the details of sample digestion methods were described in Table 1.

#### 2.1.1. Temperature Effect on Boron Recovery

In this work, the effect of heating and evaporation temperature from 65–140 °C on the digestion efficiency of this ternary mixture was studied in detail for the first time. 

By using M-method 1–M-method 3 described above, three parallel batches of the geological standard materials including W-2, JB-2a, and JR-2 were decomposed at 65, 100, and 140 °C, respectively. Here, the corresponding sample weights were 50, 50, and 25 mg (±0.5 mg), respectively. Boron was quantified by ICP-MS at masses of both ^10^B and ^11^B, and the results were given in Table 2. No obvious variations were observed between the averaged concentrations of boron measured at masses of ^10^B and ^11^B, showing boron contents in W-2, JB-2a, and JR-2 within 11.94–13.11, 29.23–29.92, and 143.8–151.4 μg/g, respectively. Apparently, there were no significant differences among the obtained boron concentrations in the studied standard materials when applying different heating and evaporation temperatures during digestion. Furthermore, the obtained boron concentrations were in good agreement with the referred values of boron (W-2: 12.5 ± 1.6 μg/g, JB-2a: 29.98 ± 0.74 μg/g, and JR-2: 145 ± 8.6 μg/g) [28,29], yielding boron recoveries for the three standard materials in the range from 95.5% to 105.5%. It can thus be deduced that this proposed digestion method could resolve the evaporation loss problem of boron during sample treatment and be tolerated with a high temperature of up to 140 °C.

#### 2.1.2. Effects of Ultrasonic Treatment on Boron Recovery

The effect of ultrasonic treatment on digestion efficiency was also investigated. By applying heating and evaporation temperature of 65 °C, another sample batch with a sample weight of 50.0 mg (±0.5 mg) was digested following M-method 4 without ultrasonic pretreatment, and sample quantification results were given in Table 2. The boron concentrations were observed to be 12.39–13.28 μg/g for W-2 and 29.75–30.08 μg/g for JB-2a. Clearly, the boron concentration from such a digestion method without ultrasonic treatment agreed with the referred values, giving boron recoveries in the range of 98.7–106.3%. This demonstrated that the 4-h ultrasonic bath was not an indispensable step and hotplate heating was sufficient in sample digestion for boron analysis. Additionally, the results of boron concentrations given at the mass of ^10^B were consistent with those obtained at the mass of ^11^B, reconfirming the indiscriminate measurements at the two boron isotopes for the studied silicate samples.

#### 2.1.3. Sample Boron Concentration Effect on Boron Recovery

In Table 2, it was found that the boron concentrations of the three parallel specimens of JR-2 treated by M-method 4 were within 143.6–148.9 μg/g when measuring at the mass of ^10^B, giving boron recoveries in the range of 99.1–102.7%. However, the boron concentrations of JR-2 measured at the mass of ^11^B were found to range from 167.4 to 173.8 μg/g, with boron recoveries up to about 120%. Such a boron concentration bias of JR-2 was obviously contrary to the results in the quantification of W-2 and JB-2a. To further study this high recovery of boron, the relationship of ^11^B signal intensity versus boron standard solutions ranging from 0–200 ng/mL was tested. Results showed that there is a good linear relationship giving a correlation coefficient *R*^2^ of 0.999. Here, it was worth noting that the sample weight in this batch was 50.0 mg (±0.5 mg), which yielded a final solution consisting of boron contents of about 625, 1500, and 7250 ng for W-2, JB-2a, and JR-2, respectively. Because there were no significant differences in boron concentrations of JR-2 obtained at masses of ^10^B and ^11^B when applying a sample weight of 25.0 mg (±0.5 mg), the boron recoveries of 115.5–119.8% for JR-2 measured at the mass of ^11^B should be ascribed to the larger sample weight containing a higher content of boron. Wilker et al. [30] reported that there existed severe carbon interference for boron determination in methyl–borate complex. Hence, the high boron level can cause high carbon contents via forming the mannitol complex in the sample solution, leading to ^12^C interference on ^11^B and thus accuracy deterioration of boron quantification. In this current study, the measurement at a mass of ^11^B equated to that at the mass of ^10^B for samples containing boron less than 3625 ng. In the case of boron content being higher than this value, the mass of ^10^B rather than ^11^B was highly recommended for boron quantification to achieve accurate results. However, a more detailed study of sample boron concentration effect on boron determination accuracy is required. 

### 2.2. A Comparison of Boron Recovery to High-Pressure Closed Acid Digestion Method

Here, the analytical efficiency including quantification accuracy and boron recovery of the laboratory high-pressure closed acid digestion method [25] was also evaluated. Table 3 showed the obtained boron concentrations of W-2, JB-2a, and JR-2 were within 8.32–8.41, 14.54–15.96, and 129.5–142.1 μg/g, respectively. The corresponding boron recoveries were found to range from 48.5% to 98.0%. Despite the remarkable loss of boron, it was clear that such a high-pressure closed digestion acid method can hold at least half of the boron in silicate samples using the binary acid mixture of HF-HNO_3_.

**Figure 1:**
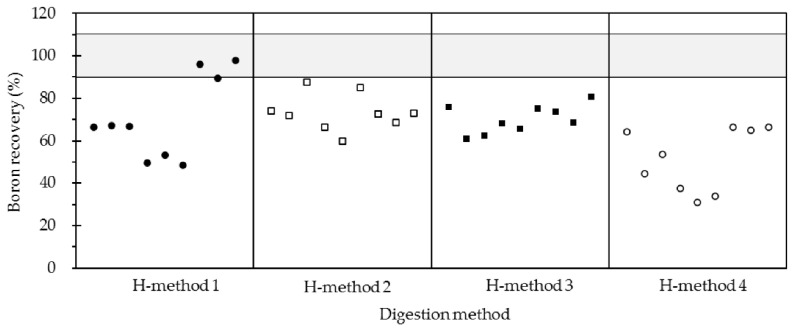
Boron recoveries of silicate samples by different high-pressure closed acid digestion methods. Here three silicate standard materials including W-2, JB-2a, and JR-2 were applied in this study. For each method, three parallel specimens of one silicate standard material with sample weight of 50 mg (±0.5 mg) were digested and the boron contents were quantified by ICP-MS at the mass of ^11^B with recoveries calculated. Every three boron recoveries for each column from left to right were the results of W-2, JB-2a, and JR-2, respectively.

By taking the volatility of formed BF_3_ into consideration, the temperature of hotplate pressure relief and evaporation was then set down to 100 or 60 °C. However, the boron recoveries for the three standard materials fell within 60.0–87.5%, which were comparable to those obtained under hotplate pressure relief and evaporation temperature of 140 °C (see Figure 1). This revealed that the temperature from 60–140 °C for this digestion method had no obvious effect on the quantification accuracy of boron. Furthermore, it was observed that the boron recoveries decreased to the range of 31.1–66.4% when excluding the hotplate pressure relief procedure. It was also found that the repetitions of parallel samples became poorer. For example, the obtained boron concentrations of W-2 were ranging from 5.57 to 8.02 μg/g, giving the RSD high as 18.1% (n = 5). Hence, it can be inferred that the boron loss of silicate samples might be caused during the high-pressure closed digestion step. Compared to the high-pressure closed digestion method, the proposed boron–mannitol complex digestion method using a ternary mixture of mannitol-HF-HNO_3_ was preferred for boron determination in silicate samples in terms of higher accuracy and full recovery of boron.

### 2.3. Long-Term Stability Study of Boron Quantification

**Figure 2:**
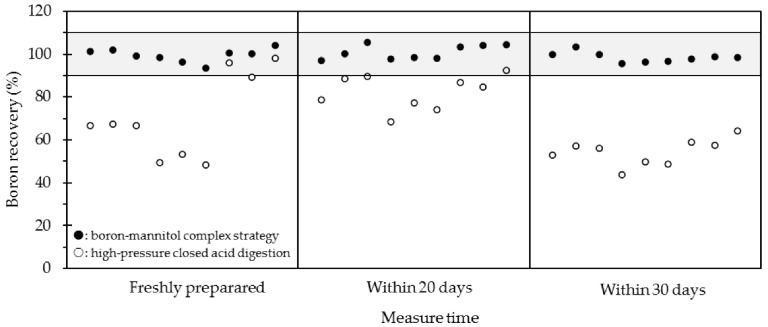
Long-term stability study of boron quantification for different digestion methods. Two batches of silicate standard materials including W-2, JB-2a, and JR-2 were decomposed using the proposed boron–mannitol complex digestion method (M-method 1) and high-pressure closed acid digestion method (H-method 1), respectively. The boron contents were repetitively measured within one month using ICP-MS at the mass of ^11^B. Every three boron recoveries for each column from left to right were the results of W-2, JB-2a, and JR-2, respectively. Here, the sample weight was 50 mg (±0.5 mg) except JR-2, which was 25 mg (±0.5 mg) when using M-method 1 as the digested method.

In this work, it was worth noting that boron contents in sample solution using a high-pressure closed acid digestion method varied within about one week, while such a phenomenon was not observed using the proposed method. To further evaluate the analytical property of this developed method, the long-term stability of boron quantification was studied. Here, two batches of the three standard silicate materials treated by M-method 1 and H-method 1 were repetitively measured at the mass of ^11^B across one month with results given in Table 4. For the M-method 1 using boron–mannitol complex strategy, it was clear that the boron concentrations were comparable, showing W-2 of 12.11–13.19, JB-2a of 28.02–29.54 and JR-2 of 141.6–151.4 μg/g, respectively, and the boron recoveries were within 93.5–104.4%. For the high-pressure closed digestion method, however, it was found that the boron contents varied with measuring time. For example, the recovery of one specimen of JR-2 changed from 95.9% to 58.9% (see Figure 2). Apparently, boron existed stable in a sample solution by this current boron–mannitol complex digestion method, making it more favorable in a geochemical laboratory, especially in the case of being impossible to measure the freshly prepared samples. However, the analytical capability of this proposed method for various silicate samples especially which contain high boron content such as tourmaline is needed in future work.

## 3. Materials and Methods

### 3.1. Instrumental Apparatus and Operating Conditions of ICP-MS

This study was carried out using an Agilent 7900 ICP-MS (Agilent, Santa Clara, CA, USA), which consisted of a concentric nebulizer, a Scott chamber with a temperature-controlled system, a standard quartz torch, an assemblage of Ni sample/skimmer cones (1.0/0.45 mm), a quadrupole mass analyzer, and a peristaltic pump. To enhance signal sensitivity, this ICP-MS equipped with a Pt shielding plate and a silicon shielding cap. 

The ICP-MS instrument worked under standard instrumental settings (1550 W of forward power, 15.0 L/min of plasma gas, 1.0 L/min of auxiliary gas, 1.05 L/min of nebulizer gas, and 10 mm of sampling depth) and was optimized daily to obtain the highest possible sensitivities for low to high mass isotopes. After the system had been subjected to warm-up for at least one hour, tunings for torch axis, EM, lens, and mass resolution/axis were accomplished using a tuning solution that contained 1.0 ng/mL of Li, Y, Ce, and U. Here, oxide formation (CeO^+^/Ce^+^) and doubly charged species (Ce^2+^/Ce^+^) were well controlled lower than 2.0%. Thereafter, the P/A factor for pulse and analog modes of the detector was calibrated using a 50 ng/mL of multi-element solution. Before quantification, a solution of digested silicate sample was introduced to flush the whole system for at least 30 min. During the measurement, a standard solution as the drift monitor was repeatedly quantified every five unknown samples. To minimize the memory effect on quantification accuracy, the system had been continuously washed in 2% HNO_3_ (*v*/*v*) solution, with a signal recovery of 25 ng/mL of the internal standard Rh checked. Boron determination was done using no gas mode of this ICP-MS. The data were read under peak jumping mode with the detector set as the dual mode and isotope dwell time fixed at 0.3 s.

### 3.2. Reagents and Chemicals 

In this work, ultrapure acids were used to reduce the procedure blanks. The commercially available acids including HNO_3_ (68% *v*/*v*, AR grade), HF (40% *v*/*v*, AR grade), and HCl (36% *v*/*v*, AR grade) were purified twice using sub-boiling distillation in Teflon stills (Savillex DST-1000-PFA, Eden Prairie, MN, USA). All solutions were prepared using ultrapure water with a resistivity of 18.2 MΩ·cm, which was produced by passing deionized water through a Milli-Q water purification system (Millipore, Bedford, MA, USA). 

The solutions used in this work were prepared by the gravimetric method. The 2% mannitol (wt.) solution was prepared by dissolving 1.0 g of mannitol (AR grade) in ultrapure water with a final solution weight of 50 g. The 8% HNO_3_ (wt.) solution and 6% HCl (wt.) solution were obtained by diluting 0.4 mL of concentrated HNO_3_ and 0.3 mL of concentrated HCl with ultrapure water to 50 g, respectively. Boron solutions with concentrations of 5, 15, 30, 50, and 100 ng/mL of boron) in 2% HNO_3_ (*v*/*v*), which were used as the external calibrators, were prepared progressively from a boron single-element standard solution of 1.0 mg/mL purchased from the National Institute of Standards and Technology, China. A solution of 2% HNO_3_ (*v*/*v*) without adding boron standard solution was utilized as the blank external calibrator. Here, to exclude any possible assay bias from long-term storage, all standard solutions were prepared freshly. 

### 3.3. Silicate Standard Materials 

Three silicate reference materials from basic to acid compositions, including diabase W-2, basalt JB-2a, and rhyolite JR-2, were selected in digestion method and long-term stability study of boron quantification in this work. The W-2 is a mafic geochemical reference material from the Geological Survey of U.S. (Reston, VA, USA), JB-2a and JR-2 are silicate standard materials from the Geological Survey of Japan (Tsukuba, Ibaraki Prefecture, Japan). 

### 3.4. Digestion Method Description

In this work, the evaporation and heating processes were finished in a boron-free ULPA filtration hood in a hundred clean room. The utilized PFA beakers were immersed in aqua regia (HNO_3_-HCl, 3:1, *v*/*v*) and ultrapure water sequentially, with each step performed at 120 °C for 24 h. Prior to usage, the labware was carefully rinsed three times with ultrapure water. The silicate samples were digested using a boron–mannitol complex strategy. Samples with a quantity of 25–50 mg (±0.5 mg) were weighed in 15 mL of PFA beakers, then 0.6 mL of concentrated HF, 30 μL of concentrated HNO_3_ and 0.5 mL of 2% mannitol were added into the samples gently. After the ternary reagents had mixed with samples, the beakers were capped tightly, and the samples were digested according to the following procedures (see Table 1). (1) The beakers were placed in an ultrasonic bath for pretreatment 4 h (M-method 1 to M-method 3). (2) The beakers were then transferred to the hotplate and heated overnight at 65 (M-method 1 and M-method 4), 100 (M-method 2), or 140 °C (M-method 3). (3) With samples evaporated to incipient dryness, 0.6 mL of 8% HNO_3_ was added, and samples were continuedly fluxed overnight. (4) Thereafter, the samples were evaporated to incipient dryness again, and 0.6 mL of 6% HCl was added with samples fluxed overnight. (5) When becoming incipiently dry, the samples were fortified with 2.0 mL of 40% HNO_3_ (*v*/*v*) and fluxed 4 h. (6) After aging overnight, the solutions were transferred to PET bottles and then gravimetrically diluted to 50 ± 0.5 g using 2% HNO_3_ (*v*/*v*) solution. Finally, the sample solutions were taken for boron quantification by ICP-MS directly.

The samples were also digested using a laboratory high-pressure closed acid method developed by Tan and Wang [25] with small modifications. In brief, 1.0 mL of HF and 0.5 mL of HNO_3_ were added in Teflon bombs with 50 ± 0.5 mg of samples. The samples were evaporated to incipient dryness, which was called hotplate pressure relief, at 140 (H-method 1), 100 (H-method 2), or 60 °C (H-method 3). Thereafter, 0.5 mL of HF and 1.0 mL of HNO_3_ were added into the samples, and the bombs were sealed and transferred into an oven at 185 °C for 12 h. After cooling, the samples were evaporated to incipient dryness at 140 (H-method 1), 100 (H-method 2), or 60 °C (H-method 3). Then, the samples were fortified with 1.0 mL of HNO_3_ and again evaporated to incipient dryness. With 2.0 mL of 40% HNO_3_ (*v*/*v*) added, the residues were re-dissolved at 135 °C for 6 h with bombs in metal jackets and then aged overnight. The final solutions were transferred to PET bottles, and then gravimetrically diluted to 50 ± 0.5 g using 2% HNO_3_ (*v*/*v*) solution. Samples were digested using H-method 4, which was similar to H-method 1 but skipped the step of hotplate pressure relief.

## 4. Conclusions

In this current work, a silicate sample digestion method using boron–mannitol complex strategy for accurate boron quantification by ICP-MS was fully investigated for the first time. With a ternary mixture of mannitol-HF-HNO_3_ as the digestion reagent, about 50 mg of silicate samples can be completely decomposed. It was found that the results had no significant differences under the digestion temperature from low to high of 65–140 °C, and the boron concentrations of the studied silicate standard materials were in good agreement with the referred values, showing indiscriminate measurements at masses of ^10^B and ^11^B when sample solution consisted of boron less than 3625 ng. However, there was severe ^12^C interference on ^11^B causing accuracy deterioration due to high carbon contents from the formed boron–mannitol complex when sample boron content was 7250 ng. Thus, the isotope of ^10^B rather than ^11^B was recommended for boron quantification to achieve accurate results. The comparison of the proposed method and the laboratory high-pressure closed digestion method showed that boron was more stable in the form of boron–mannitol complex within one month and yielded boron recoveries of 93.5–104.4%. By applying this developed digestion method, which was characterized by complete decomposition capability, full recovery of boron, and long-term quantification stability of boron, the boron concentrations in silicate standard samples were successfully determined. This current work for accurate determination of boron in silicate samples was of great value in providing critical data for further isotope analyses and promising the extensive application of boron in the study of the provenance identification and associated geological activities.

## Figures and Tables

**Table 1:** Different sample digestion methods based on boron–mannitol complex.

Procedure	Method			
	M-Method 1	M-Method 2	M-Method 3	M-Method 4
Ultrasonic pretreatment	Add 0.6 mL of HF, 30 μL of HNO_3_, and 50 μL of 2% mannitol into 25–50 mg of sample			
	4 h	4 h	4 h	–
Hotplate digestion	65 °C, overnight	100 °C, overnight	140 °C, overnight	65 °C, overnight
Fluoride formation prevention	Dry at 65 °C, then add 0.6 mL of 8% HNO_3_ and flux overnight	Dry at 100 °C, then add 0.6 mL of 8% HNO_3_ and flux overnight	Dry at 140 °C, then add 0.6 mL of 8% HNO_3_ and flux overnight	Dry at 65 °C, then add 0.6 mL of 8% HNO_3_ and flux overnight
Fluoride decomposition	Dry at 65 °C, then add 0.6 mL of 6% HCl and flux overnight	Dry at 100 °C, then add 0.6 mL of 6% HCl and flux overnight	Dry at 140 °C, then add 0.6 mL of 6% HCl and flux overnight	Dry at 65 °C, then add 0.6 mL of 6% HCl and flux overnight
Hotplate redissolution	Dry at 65 °C, then add 2.0 mL of 40% HNO_3_ and flux 4 h	Dry at 100 °C, then add 2.0 mL of 40% HNO_3_ and flux 4 h	Dry at 140 °C, then add 2.0 mL of 40% HNO_3_ and flux 4 h	Dry at 65 °C, then add 2.0 mL of 40% HNO_3_ and flux 4 h
Sample solution for ICP-MS	Age overnight, and dilute 1000-fold using 2% HNO_3_			

**Table 2:** Boron determination results with boron–mannitol complex digestion methods.

Method	Sample	^10^B			^11^B		
		Content ^1^ μg/g	2σ	Recovery ^2^ %	Content μg/g	2σ	Recovery %
M-method 1	W-2_1	11.94	0.20	95.5	12.11	0.12	96.9
	W-2_2	12.33	0.18	98.7	12.51	0.17	100.1
	W-2_3	13.10	0.20	104.8	13.19	0.12	105.5
	JB-2a_1	29.46	0.16	98.3	29.29	0.16	97.7
	JB-2a_2	29.92	0.39	99.8	29.54	0.20	98.5
	JB-2a_3	29.32	0.21	97.8	29.41	0.19	98.1
	JR-2_1	148.1	0.6	102.2	149.8	1.0	103.3
	JR-2_2	149.4	5.3	103.1	150.9	5.7	104.0
	JR-2_3	149.6	0.8	103.2	151.4	0.7	104.4
M-method 2	W-2_1	13.07	0.33	104.6	13.11	0.16	104.9
	W-2_2	12.81	0.20	102.5	12.89	0.12	103.1
	W-2_3	12.78	0.11	102.2	12.81	0.12	102.5
	JB-2a_1	29.74	0.26	99.2	28.84	0.28	96.2
	JB-2a_2	29.61	0.42	98.8	28.71	0.11	95.8
	JB-2a_3	29.37	0.13	98.0	28.77	0.17	96.0
	JR-2_1	145.2	1.1	100.1	144.4	1.5	99.6
	JR-2_2	146.1	1.3	100.8	144.7	1.0	99.8
	JR-2_3	145.8	1.4	100.5	145.8	0.4	100.5
M-method 3	W-2_1	12.31	0.21	98.5	12.46	0.19	99.6
	W-2_2	12.20	0.20	97.6	12.60	0.08	100.8
	W-2_3	12.25	0.17	98.0	12.43	0.08	99.5
	JB-2a_1	29.30	0.39	97.7	29.55	0.26	98.6
	JB-2a_2	29.23	0.31	97.5	29.51	0.14	98.4
	JB-2a_3	29.51	0.37	98.4	29.90	0.24	99.7
	JR-2_1	143.8	0.8	99.2	144.5	0.4	99.7
	JR-2_2	145.5	0.7	100.4	146.7	0.6	101.2
	JR-2_3	144.8	1.0	99.9	146.1	0.9	100.8
M-method 4	W-2_1	12.39	0.21	99.1	12.48	0.09	99.9
	W-2_2	13.01	0.14	104.1	12.95	0.06	103.6
	W-2_3	13.28	0.17	106.3	13.27	0.07	106.1
	JB-2a_1	29.94	0.28	99.9	29.58	0.24	98.7
	JB-2a_2	30.08	0.21	100.3	29.75	0.20	99.2
	JB-2a_3	29.95	0.25	99.9	29.82	0.15	99.5
	JR-2_1	143.6	1.1	99.1	167.4	2.5	115.5
	JR-2_2	145.6	1.3	100.4	172.5	1.8	118.9
	JR-2_3	148.9	1.0	102.7	173.8	1.1	119.8

^1^ Result was the average of five individual measurements and given as 95% confidential intervals. ^2^ Recovery was calculated using the form of (*Boron*_masured_/*Boron*_referred_) × 100%.

**Table 3:** Results of boron determination with high-pressure closed digestion method.

Method	Specific Procedures		Sample	Content ^1^ μg/g	2σ	Recovery %
H-method 1	Hotplate pressure relief at 140 °C	Evaporation at 140 °C	W-2_1	8.32	0.05	66.6
			W-2_2	8.41	0.13	67.2
			W-2_3	8.33	0.12	66.7
			JB-2a_1	14.84	0.11	49.5
			JB-2a_2	15.96	0.12	53.2
			JB-2a_3	14.54	0.17	48.5
			JR-2_1	139.0	0.6	95.9
			JR-2_2	129.5	0.6	89.3
			JR-2_3	142.1	1.2	98.0
H-method 2	Hotplate pressure relief at 100 °C	Evaporation at 100 °C	W-2_1	9.28	0.08	74.2
			W-2_2	8.96	0.10	71.7
			W-2_3	10.94	0.19	87.5
			JB-2a_1	19.91	0.16	66.4
			JB-2a_2	17.97	0.10	60.0
			JB-2a_3	25.54	0.28	85.2
			JR-2_1	105.2	1.0	72.6
			JR-2_2	99.71	0.52	68.8
			JR-2_3	105.6	0.5	72.8
H-method 3	Hotplate pressure relief at 60 °C	Evaporation at 60 °C	W-2_1	9.48	0.07	75.8
			W-2_2	7.60	0.29	60.8
			W-2_3	7.81	0.03	62.4
			JB-2a_1	20.44	0.05	68.2
			JB-2a_2	19.69	0.04	65.7
			JB-2a_3	22.55	0.08	75.2
			JR-2_1	107.0	1.1	73.8
			JR-2_2	99.60	1.34	68.7
			JR-2_3	116.7	1.0	80.5
H-method 4	–	Evaporation at 140 °C	W-2_1	8.02	0.10	64.1
			W-2_2	5.57	0.07	44.6
			W-2_3	6.69	0.09	53.5
			JB-2a_1	11.22	0.08	37.4
			JB-2a_2	9.31	0.08	31.1
			JB-2a_3	10.18	0.08	34.0
			JR-2_1	96.24	0.79	66.4
			JR-2_2	94.00	1.20	64.8
			JR-2_3	96.25	0.53	66.4

^1^ Result was the average of five individual measurements and given as 95% confidential intervals.

**Table 4:** Results of long-term stability study of boron by ICP-MS.

Method ^1^	Proposed boron–mannitol complex digestion method								
Sample	Freshly prepared			Within 20 days			Within 30 days		
	Content ^2^ μg/g	2σ	Recovery %	Content μg/g	2σ	Recovery %	Content μg/g	2σ	Recovery %
W-2_1	12.66	0.24	101.3	12.11	0.12	96.9	12.47	0.20	99.8
W-2_2	12.75	0.43	102.0	12.51	0.17	100.1	12.94	0.10	103.5
W-2_3	12.39	0.33	99.1	13.19	0.12	105.5	12.50	0.14	100.0
JB-2a_1	29.54	0.46	98.5	29.29	0.16	97.7	28.66	0.17	95.6
JB-2a_2	28.85	0.44	96.2	29.54	0.20	98.5	28.92	1.54	96.5
JB-2a_3	28.02	0.45	93.5	29.41	0.19	98.1	28.99	0.29	96.7
JR-2_1	145.8	2.5	100.5	149.8	1.0	103.3	141.6	0.8	97.6
JR-2_2	145.5	1.1	100.3	150.9	5.7	104.0	143.2	0.7	98.7
JR-2_3	151.0	1.0	104.1	151.4	0.7	104.4	142.6	1.7	98.4
Method	High-pressure closed digestion method								
Sample	Freshly prepared			Within 20 days			Within 30 days		
	Content μg/g	2σ	Recovery %	Content μg/g	2σ	Recovery %	Content μg/g	2σ	Recovery %
W-2_1	8.32	0.05	66.6	9.84	0.08	78.8	6.63	0.12	53.1
W-2_2	8.41	0.13	67.2	11.06	0.12	88.5	7.17	0.09	57.3
W-2_3	8.33	0.12	66.7	11.19	0.18	89.5	7.00	0.13	56.0
JB-2a_1	14.84	0.11	49.5	20.49	0.16	68.4	13.08	0.14	43.6
JB-2a_2	15.96	0.12	53.2	23.13	0.24	77.1	14.89	0.15	49.7
JB-2a_3	14.54	0.17	48.5	22.19	0.19	74.0	14.61	0.16	48.7
JR-2_1	139.0	0.6	95.9	125.6	1.1	86.6	85.35	0.56	58.9
JR-2_2	129.5	0.6	89.3	122.6	1.4	84.6	83.58	0.40	57.6
JR-2_3	142.1	1.2	98.0	134.1	1.1	92.5	93.19	0.71	64.3

^1^ M-method 1 and H-method 1 were applied as sample digestion methods. ^2^ Result was the average of five repetitive measurements and given as 95% confidential intervals.

## Data Availability

Not applicable.

## Sample Availability

Samples of the silicate standard materials are available from the authors.
